# A Heavy Brain

**Published:** 1882-12

**Authors:** 


### A HEAVY BRAIN.

The chief interest in this case lies in the great weight of the brain, and its high specific gravity, in relation to the highly gifted intellectual power exhibited by the individual during life. As this brain weighed very nearly 60 ounces, it exceeds that of all others usually quoted, with the exception only of Cuvier's, which weighed 64½ ounces, and that of Dr. Abercrombie, which weighed 63 ounces. Sir J. N. Simpson's brain weighed 54 ounces, and that of Agassiz 53.4 ounces. It is well known that the average weight of the adult male brain is under 50 ounces. The specific gravity of the brain I examined was 1.049, and this is as high as any recorded. From

Professor Aitken's work I find that the average specific gravity of the brain is 1.036, and the highest specific gravity of the densest part of a brain ever taken by Professor Aitken, or any one else, I believe, is 1.049.

The weight of the brain in this case was, in the first instance, taken by the orderly corporal in charge of our microscope, and recorded by him on the blackboard in the mortuary. I immediately verified its accuracy by weighing the organ myself, and also verified the correctness of the weighing-machine. The specific gravity was taken very carefully. Surgeon-Major Hogg, Army Medical Department, was present at the time.

The average cranial capacity of the adult male head is, I believe, about 90 cubic inches. Cuvier's is reported to have been about 118. In the case which I now record it must have been about 108. "*From Brain-Weight and Brain-Power*," *by* Dr. J. P. H. Boileau, *in Popular Science Monthly for December.*

